# The Relationship between Serum Ferritin and Insulin Resistance in Different Glucose Metabolism in Nonobese Han Adults

**DOI:** 10.1155/2015/642194

**Published:** 2015-08-19

**Authors:** Bo-wei Liu, Xu-min Xuan, Jun-ru Liu, Fang-ning Li, Fu-Zai Yin

**Affiliations:** Department of Endocrinology, The First Hospital of Qinhuangdao, No. 258 Wenhua Road, Qinhuangdao, Hebei 066000, China

## Abstract

The exact mechanism through which elevated serum ferritin promotes the development of type 2 diabetes is unknown. This study showed that ferritin concentration in impaired glucose regulation and newly diagnosed diabetes mellitus subjects of nonobesity already significantly increased when compared with normal glucose tolerant subjects of nonobesity. Elevated serum ferritin levels are associated with insulin resistance and may be not associated with the decline of insulin beta cells in different status of glucose tolerance in nonobese Han adults.

## 1. Introduction

Recent studies reported the prevalence of diabetes in China soared from 1.9 to 11.6% between 1993 and 2010 [1, 2]. The prevalence of prediabetes is rising which is an important risk factor for the development of overt diabetes and cardiovascular disease. China has to face the major public health problem and economic burden of potential diabetic patients. Thus, clarifying its etiology and looking for modifiable risk factors are of paramount importance for diabetes control and prevention.

Iron is an important mineral in normal physiological processes, and ferritin is a specialized iron storage protein, which reflects iron stores in the body [3]. Serum ferritin (SF) has been found to be a reliable tool, providing that confounding effects by inflammatory, hepatic, or neoplastic diseases are excluded [4]. It has been used as a surrogate variable to reflect body iron stores in healthy individuals. Previous studies have demonstrated an association between increased SF levels and higher risks of diabetes [5, 6]. However, the exact mechanism through which elevated SF promotes the development of type 2 diabetes is unknown.

Obesity has become a major global health problem and is associated with the risk of type 2 diabetes and insulin resistance [7]. In recent years, a number of studies have investigated the association between SF levels and various types of adiposity [8]. Is serum ferritin associated with insulin resistance or islet *β* cell function when the interference factor of obesity has been ruled out? Therefore, in the present study, we aimed to investigate the changes of ferritin metabolism in nonobese Han adults with different status of glucose tolerance and the relationship between serum ferritin (SF) and insulin resistance.

## 2. Methods

### 2.1. Subjects

The 96 (male/female, 62/34) (aged 49.8 ± 11.9 years) subjects were recruited from the First Hospital of Qinhuangdao for health examinations that BMI was normal and undiagnosed diabetes in Qinhuangdao, Hebei province during 2011. Each participant will accept a questionnaire. The inclusion criteria included the following: (1) subjects were clinically stable with no previous medical history of diabetes, hypertension, dyslipidemia, coronary artery diseases, or cerebral stroke; (2) subjects were without clinical evidence of endocrinopathy; (3) subjects were not taking medications known to affect glucose and lipid metabolism, such as statins, glucocorticoids, thyroid hormones, and thiazide diuretics; (4) subjects were not neoplasia and liver disease; (5) female subjects were postmenopausal. The exclusion criteria included the following: (1) subjects with hepatic or renal dysfunction (>1.5-fold elevation of alanine aminotransferase, aspartate aminotransferase, or serum creatinine >115 *μ*mol/L) and (2) subjects with anemia, blood transfusion, and the recent use of iron, who smokers, consuming alcohol, and having acute and chronic inflammation. This study was approved by the ethics committee of the First Hospital of Qinhuangdao. All subjects provided written informed consent before study initiation.

### 2.2. Anthropometric Measurements

Anthropometric measurements, including height, weight, waist circumference (WC), and blood pressure, were obtained while the subjects were in light clothing and not wearing shoes. Body mass index (BMI) was calculated by dividing weight (kg) by height squared (m^2^). Blood pressure was measured twice with a mercury sphygmomanometer after 10 min of rest while the subjects were seated, and the average of the two measurements was used for analysis.

### 2.3. Laboratory Examinations

After an overnight fast of 8 h, blood samples were drawn from an antecubital vein in each subject into vacutainer tubes. All subjects underwent oral glucose tolerance test (OGTT) with 75 g of oral anhydrous glucose at 8:00 AM after 8 h of fasting. 75 g anhydrous glucose was dissolved in 250 mL water. Peripheral venous blood samples were taken at 0, 1, 2, and 3 hours after glucose loading. Fasting plasma glucose (FPG) concentration and 2 h plasma glucose (2-hPG) of OGTT concentration were measured using the glucose oxidase method, and serum lipid levels were measured using enzymatic assays with an autoanalyzer (Hitachi, Tokyo, Japan). Plasma concentrations of fasting true insulin (FTI) and 2 h insulin of OGTT were measured by enzyme linked immunosorbent assay (ELISA) with model 680 microplate reader (BIO-RAD, America). The ELISA kits were purchased from USCNLIFE company, America (intra-assay < 3% and interassay < 4%). Plasma concentrations of SF were measured by electrochemiluminescence with model Elecsys 2010 immunity analyzer (Roche, German). The kits were purchased from Roche company, German (intra-assay < 3% and interassay < 5%). The following equation was used to calculate the homeostasis model assessment (HOMA-) IR index (fasting insulin level × fasting glucose level)/22.5 and (HOMA-) *β* index (20 × fasting insulin level)/(fasting glucose level − 3.5) [9–11].

### 2.4. Definition of Groups

Glucose metabolism classification using 2008 American diabetes association recommended standards. The definition of nonobese is BMI < 25 kg/m^2^, and it was identified by WHO (1999) criteria.

The subjects were divided into three groups according to the result of OGTT with 75 g oral anhydrous glucose. The cases and controls were age- and gender-matched. The group NGT consisted of 32 normal glucose tolerant subjects (male/female 19/13, age 49.3 ± 10.9 years), group IGR consisted of 33 impaired glucose tolerant and/or impaired fasting glucose subjects (male/female 22/11, age 50.1 ± 11.1 years), and group DM consisted of 31 newly diagnosed diabetes mellitus subjects (male/female 21/10, age 49.8 ± 11.2 years).

### 2.5. Statistical Analyses

All analyses were performed using the SPSS 14.0 statistical software (SPSS 14.0 for Windows; SPSS, Inc., Chicago, IL). Values were expressed as mean with standard deviation. Count data was expressed as percentage. When not normally distributed, the data were ln-transformed for analysis and are expressed as medians with interquartile ranges. Comparisons were done between the three groups using analysis of variance (ANOVA) and Pearson chi-square analysis. Multiple comparisons were made by least significant difference (LSD) test. Correlation analysis was made by Spearman test. To examine the association between serum ferritin and other variables, multiple linear regression analyses were tested. *P* < 0.05 was considered statistically significant.

## 3. Results

Anthropometric and biochemical data are presented in Table 1. The age (*X* ^2^ = 0.756, *P* = 0.913) and gender (*X* ^2^ = 0.541, *P* = 0.73) were similar in three groups (*P* > 0.05). Diastolic blood pressure (DBP), systolic blood pressure (SBP), FPG, 2-hPG, triglycerides (TG), SF, and HOMA-IR were all significantly higher and high density lipoprotein cholesterol (HDL-C) was significantly lower in the DM subjects than IGR and NGT subjects (*P* < 0.05). FTI was decreased in the DM subjects than IGR subjects (*P* < 0.05). FPG, 2-hPG, FTI, TG, SF, and HOMA-IR were all significantly higher in the IGR subjects than NGT subjects (*P* < 0.05). HOMA-*β*, 2 h insulin were decreased and WC was increased in the DM and IGR subjects than NGT subjects (*P* < 0.05).

After adjustment for age and gender of the partial correlation analysis showed that SF positively correlated with FPG (*r* = 0.451, *P* = 0.000), FTI (*r* = 0.455, *P* = 0.000), TG (*r* = 0.383, *P* = 0.003), and HOMA-IR (*r* = 0.482, *P* = 0.000) (Table 2). When HOMA-IR was considered as the dependent variables in a multiple regression analysis with age, sex, WC, SBP, DBP, 2-hPG, 2 h insulin, TG, HDL-C, and SF as independent variables, SF (*β* = 12.13, *P* = 0.023) and TG (*β* = 0.102, *P* = 0.041) maintained independent association with HOMA-IR (Table 3). When HOMA-*β* was considered as the dependent variables in a multiple regression analysis with age, sex, WC, SBP, DBP, 2-hPG, 2 h insulin, TG, HDL-C, and SF as independent variables, 2-hPG (*β* = −0.101, *P* = 0.013) maintained independent association with HOMA-*β* (Table 4).

## 4. Discussion

In the present study, we found that the SF levels significantly increased in nonobese adults of China when the glucose metabolic disorder was deteriorated. Our results showed that, in different glucose metabolism, the increased SF levels were associated with the increase of FPG, blood pressure, triglycerides, and HOMA-IR. However, compared with increased SF levels, HDL-C level was lower in different glucose metabolism in nonobese adults. It is well known that obesity, hyperglycemia, hypertension, and dyslipidemia are the main components of the metabolic syndrome. Our results further illustrated that abnormal glucose metabolism, hypertension, dyslipidemia, insulin resistance, and high SF levels were synchronous when the interference factors of obesity were avoided. In our study, there was positive correlation between FPG, FTI, TG, HOMA-IR, and SF levels. In multiple linear regression analysis showed that elevated SF levels and TG were significant independent predictors for HOMA-IR. However, 2 h plasma glucose was significant independent predictor for HOMA-*β*. Our findings provided us with new evidence of SF being regarded as a biomarker for insulin resistance but not relevant with beta cell function.

Several studies showed the association between SF and diabetes, and most of them were based on Caucasian samples [12–14]. However, the observation in North Indians was in sharp contrast to the earlier studies published from the West stressing that SF was increased in obesity and diabetes. They conclude that SF may not be a strong risk factor in the pathogenesis of obesity and diabetes [15]. In 2014, a cross-sectional survey of 8,235 participants in china reported that elevated SF levels were associated with higher risks of diabetes, higher levels of HbA1c, and HOMA-IR independent of several confounders [16]. Recent studies reported higher SF levels were associated with diabetes incidence in an elderly Chinese population [6] and Korean men [17]. Our results are consistent with the above research.

Of course the exact mechanism of the association between SF and diabetic disorder remains to be clarified. So far, several possible biological pathways might be proposed to explain the observed findings. Firstly, SF is regarded as a biomarker of body iron store, whose catalytic effects could induce lipid peroxidation [18]. Lipid peroxidation might be involved in the development of insulin resistance [19]. Secondly, excess body iron may be directly involved in insulin signaling [20] and is able to form highly reactive free radicals, which can lead to disturbed glucose metabolism and subsequent hyperglycemia [21]. Finally, there are some evidences of a relevant relationship between SF levels and inflammation [22]. In addition, hepcidin, a peptide made in the liver, is elevated in response to hypoxia or inflammation and is correlated with increases in ferritin. It has been suggested that leptin seems to induce the expression of hepcidin via the JAK2/STAT3 pathway [23, 24].

Admittedly, our study had some limitations that deserved to be considered when interpreting the results. First, our study was that this research is a cross-sectional study. We cannot determine the causal relationship between SF and glucose metabolism in nonobese Han adults. Experimental and prospective studies are warranted to elucidate the role of SF in glucose metabolism. Second, SF levels differ significantly according to sex. Many of the research conclusion showed that gender might modify the effects of SF on diabetes and insulin resistance [5, 16, 25–27]. This might be merely statistical interaction, which does not mean biological interaction between gender and ferritin. Therefore, the longitudinal relationship study between SF levels and glucose metabolism in nonobese Han adults needs to be confirmed.

In conclusion, ferritin concentration in IGR and DM subjects of nonobese already significantly increased when compared with NGT subjects of nonobesity. Elevated SF levels are associated with insulin resistance and may be not associated with the decline of insulin beta cells in different status of glucose tolerance in nonobese Han adults.

## Figures and Tables

**Table 1:** Clinical and laboratory characteristics of the subjects in different groups.

Variable	NGT	IGR	DM	*F*	*P*
	*n* = 32	*n* = 33	*n* = 31		
BMI (kg/m^2^)	22.7 ± 1.2	23.1 ± 1.1	23.2 ± 1.1	1.186	0.232
WC (cm)	77.4 ± 6.7	82.5 ± 5.6^▲^	83.3 ± 7.7^▲^	5.020	0.009
SBP (mmhg)	118.2 ± 14.4	124.9 ± 18.2	140.0 ± 27.9^▲■^	6.164	0.004
DBP (mmhg)	76.4 ± 10.4	77.4 ± 9.4	89.3 ± 10.4^▲■^	10.918	0.000
FPG (mmol/L)	5.3 ± 0.2	6.0 ± 0.5^▲^	7.6 ± 0.6^▲■^	137.08	0.000
OGTT, 2 hPG	6.3 ± 0.3	8.2 ± 0.6^▲^	11.3 ± 0.6^▲■^	161.11	0.000
FTI (*μ*IU/mL) (IQR)	7.8 ± 3.6	11.6 ± 4.9^▲^	8.3 ± 3.7^■^	5.714	0.005
OGTT, 2 h insulin (IQR)	43.6 ± 6.6	32.2 ± 4.1^▲^	28.7 ± 4.6^▲^	19.724	0.000
TG (mmol/L) (IQR)	1.1 (0.5, 1.5)	1.0 (0.6, 3.9)^▲^	1.3 (0.5, 3.1)^▲■^	16.78	0.000
HDL-C (mmol/L)	1.5 ± 0.2	1.5 ± 0.4	1.1 ± 0.2^▲■^	13.907	0.000
HOMA-IR (IQR)	2.4 ± 0.8	3.3 ± 1.1^▲^	4.9 ± 2.6^▲■^	17.324	0.000
HOMA-*β* (IQR)	121.3 ± 36.7	97.2 ± 42.3^▲^	89.6 ± 46.4^▲^	2.861	0.048
SF (*μ*g/mL) (IQR)	1.8 ± 0.2	2.0 ± 0.3^▲^	2.2 ± 0.2^▲■^	14.55	0.000

Values are expressed as mean (SD), and when not normally distributed, they were ln-transformed for analysis and are expressed as medians (IQR). SD: standard deviation; IQR: interquartile range. Comparisons were done between the three groups using analysis of variance (ANOVA) and Kruskal-Wallis. If there was statistically significant, multiple comparisons were made by least significant difference (LSD) test. Group NGT consisted of 32 normal glucose tolerant subjects; group IGR consisted of 33 impaired glucose tolerant and/or impaired fasting glucose subjects; group DM consisted of 31 newly diagnosed diabetes mellitus subjects. BMI: body mass index; WC: waist circumference; SBP: systolic blood pressure; DBP: diastolic blood pressure; SF: serum ferritin; FPG: fasting plasma glucose; OGTT: oral glucose tolerance test; 2 hPG: 2 h plasma glucose; FTI: fasting true insulin; TG: triglycerides; HDL-C: high density lipoprotein cholesterol; HOMA: homeostasis model assessment; HOMA-IR: homeostasis model assessment of insulin resistance; HOMA-*β*: homeostasis model assessment of beta cell function. ^▲^Compared with group NGT *P* < 0.05,  ^■^compared with group IGR *P* < 0.05.

**Table 2:** Simple correlations between serum ferritin and other variables in the study subjects (after adjustment for age and gender).

Variable	*r*	*P*
Waist circumference (cm)	0.084	0.528
Systolic blood pressure (mmHg)	−0.023	0.863
Diastolic blood pressure (mmHg)	0.099	0.454
Triglyceride (mmol/L)	0.383	0.003
High density lipoprotein cholesterol (mmol/L)	−0.295	0.023
Fasting plasma glucose (mmol/L)	0.451	0.000
Oral glucose tolerance test, 2 h plasma glucose (mmol/L)	0.179	0.233
Fasting insulin (*μ*IU/mL)	0.455	0.000
Oral glucose tolerance test, 2 h insulin (*μ*IU/mL)	0.215	0.151
HOMA-IR	0.482	0.000
HOMA-*β*	0.171	0.197

**Table 3:** Multiple linear regression analyses for HOMA-IR (stepwise method).

Model	Unstandardized coefficients *B*	Std. error	Standardized coefficients *B*	*t*	*P*	95% CI	*R* ^2^
(Constant)	126.342	12.462		9.437	0.000	87.783 to 152.362	
Serum ferritin	12.13	6.018	0.134	3.153	0.023	4.102 to 27.653	0.204
Triglyceride	0.102	0.023	0.103	1.865	0.041	0.009 to 0.168	0.229

Dependent variable: HOMA-IR: homeostasis model assessment of insulin resistant.

**Table 4:** Multiple linear regression analyses for HOMA-*β* (stepwise method).

Model	Unstandardized coefficients *B*	Std. error	Standardized coefficients *B*	*t*	*P*	95% CI	*R* ^2^
(Constant)	87.423	9.514		4.743	0.000	583.77 to 162.532	
OGTT, 2 h plasma glucose	−0.101	0.003	−0.034	−1.253	0.013	−0.402 to −0.009	0.104

Dependent variable: HOMA-*β*: homeostasis model assessment of beta cell function.
